# Deep learning hybrid model for analyzing and predicting the impact of imported malaria cases from Africa on the rise of *Plasmodium falciparum* in China before and during the COVID-19 pandemic

**DOI:** 10.1371/journal.pone.0287702

**Published:** 2023-12-06

**Authors:** Eric Kamana, Jijun Zhao

**Affiliations:** Complexity Science Institute, Qingdao University, Qingdao, China; Jeonbuk National University, REPUBLIC OF KOREA

## Abstract

**Background:**

*Plasmodium falciparum* cases are rising in China due to the imported malaria cases from African countries. The main goal of this study is to examine the impact of imported malaria cases in African countries on the rise of *P*. *falciparum* cases in China before and during the COVID-19 pandemic.

**Methods:**

A generalized regression model was used to investigate the association of time trends between imported malaria cases from 45 African countries and *P*. *falciparum* cases in 31 provinces of China from 2012 to 2018 before the COVID-19 pandemic and during the COVID-19 pandemic from October 2020 to May 2021. Based on the analysis, we proposed a statistical and deep learning hybrid approach to model the resurgence of malaria in China using monthly data of *P*. *falciparum* from 2004 to 2016. This study builds a hybrid model known as the ARIMA-GRU approach for modeling the *P*. *falciparum* cases in all provinces of China and the number of malaria deaths in China before and during the COVID-19 pandemic.

**Results:**

The analysis showed an emerging link between the rise of imported malaria cases from Africa and *P*. *falciparum* cases in many provinces of China. Many imported malaria cases from Africa were *P*. *falciparum* cases. The proposed deep learning model achieved a high prediction accuracy score on the testing dataset of 96%.

**Conclusion:**

The study provided an analysis of the reduction of *P*. *falciparum cases* and deaths caused by imported *P*. *falciparum* cases during the COVID-19 pandemic due to the control measures regarding the limitation of international travel in China. The Chinese government has to prepare the imported malaria control measures after the normalization of international travel, to prevent the resurgence of malaria disease in China.

## Background

Recently, the malaria species that has the most cases imported from endemic countries is *Plasmodium falciparum* (*P*. *falciparum*). *P*. *falciparum* remains problematic in malaria-free countries because of its difficult diagnosis, extensive treatment burden, relatively high mortality rate, and potential to spread locally [1]. Malaria disease imported from Africa has been a common problem in non-endemic countries like the UK and France over the past decades. Some of these countries have historical, linguistic, and cultural ties to Africa, and certain demographic groups have higher infection rates, including travelers visiting relatives and friends in endemic African countries [1–3].

*P*. *falciparum* is responsible for most of the morbidity and mortality of humans in sub-Saharan Africa and other malarious tropical areas in the world. It is also responsible for 83.78% of the imported malaria cases in the provincial capital of the Hubei province, Wuhan, from 2011 to 2018 [4]. *Plasmodium falciparum* malaria has been increasingly imported from Africa to China due to Chinese overseas investment and labor-related Chinese travelers. Since 2011 to 2015, 8653 *P*. *falciparum* cases and 98 deaths (11.3 per 1000 cases) were imported from 41 sub-Saharan countries into China, with most cases (91.3%) occurring in Chinese laborers [5]. *P*. *falciparum* cases have been remarkably narrowed to the provinces of Yunnan and Hainan [6]. However, the importation of *P*. *falciparum* malaria has increased in recent years with a higher fatality rate, which poses a threat to the health of labor travelers and challenges to China’s healthcare system. Additionally, it would increase the risk of malaria being reintroduced and spread to malaria-free receptive areas [6–10].

Africa to China recently is an emerging route of *P*. *falciparum* infection movements by Chinese workers returning home from Africa countries [8]. This route has risen over the past decade due to the increased Chinese investment in African countries and the increased number of workers exported from China to Africa [5]. Imported malaria cases are detected daily by an established surveillance system in China. However, there are still vectors out there capable of transmitting an imported malaria case that may go undetected by the system. Because of the changes in the environment, climate, and anti-malaria interventions, the distribution of *Anopheles* vectors like *Anopheles Sinensis* (*An*. *Sinensis*) had changed over the same period as *Plasmodium* species. The interaction between the imported malaria cases and the mosquito vectors may result in malaria transmission in China, potentially increasing the risk of malaria resurgence.

Early diagnosis and control of imported malaria have been particularly difficult since COVID-19 (coronavirus disease 2019) emerged in December of 2019 since there are similarities between malaria clinical symptoms and COVID-19 in its early stages. While malaria and COVID-19 can have similar presentations, common symptoms they share include but are not limited to: fever, breathing difficulties, tiredness, and acute onset headache, which may lead to misdiagnosis of malaria for COVID-19 and vice versa, particularly when the clinician relies mainly on symptoms. Entry personnel from malaria-affected areas of African countries were subject to surveillance for malaria. There were at least 100,000 tests conducted in the Guangdong Province; 154 confirmed malaria cases occurred during this surveillance. This surveillance identified that *P*. *falciparum* has a high percentage of the malaria species. During COVID-19, the imported malaria cases in Guangdong province were from 23 African countries [11]. Under the situation of COVID-19 pandemic, two imported malaria cases from African countries were diagnosed in Beijing province. The first case was returned in Beijing from Malawi in March 2020. The second case was returned in October 2020 and diagnosed with malaria during the isolation period for COVID-19 [12].

In the twenty-first century, international traffic has been cited as a major contributor to the increase of imported malaria diseases. Since 2010, autochthonous malaria was eliminated in most of the provinces of China, and only imported cases started to highly increase two years later. Following the lockdown of Wuhan due to COVID-19 spreading, almost all countries have limited international travel and implemented border controls as a global response to the crisis. The stagnation of traffic during the COVID-19 pandemic has significantly decreased the number of imported malaria cases in China. Nevertheless, after the lockdown of many provinces of China due to COVID-19, the number of imported malaria from African countries has remarkably decreased [13]. However, imported malaria cases may increase after the normalization of international traffic, due to the association between imported malaria and international traffic. A resurgence of malaria has been caused in several countries by the importation of malaria cases, which indicates how important the importation of malaria is to re-emergence. To examine the decrease of *P*. *falciparum* cases in China due to the stagnation of international traffic during COVID-19, our study analyzed the association between imported malaria cases from African countries and the number of *P*. *falciparum* cases in China before and during the COVID-19 pandemic. Our goals are to demonstrate how imported malaria cases from African countries influenced the number of *P*. *falciparum* cases and the impact of the COVID-19 pandemic on the decline of *P*. *falciparum* from China. To demonstrate the impact of imported malaria cases from African countries on the trends of *P*. *falciparum* cases in China, we predicted the number of *P*. *falciparum* cases in each province of China using a hybrid deep learning model and forecast the number of malaria deaths in China caused by imported *P*. *falciparum* cases imported before and during the COVID-19 pandemic.

Few studies in health care have applied hybrid deep learning approaches to predict infectious diseases. Rahmadani et Lee (2020) proposed a hybrid model to predict COVID-19 by combining the LSTM neural network model and the epidemic meta-model in South Korea. The combined deep learning model generates more accurate modelling parameters, which are used for epidemic meta-population modeling. To test the effectiveness of the proposed deep learning hybrid approach, the study used COVID-19 data, and the prediction was compared with other single deep learning prediction models. The analysis shows that the proposed hybrid approach outperforms other teste deep learning models and is expected to contribute to the effective control of COVID-19 pandemic [14]. Infectious diseases are highly emerged, it is important to build a forecasting model and put in action control measures that restrain their exploding. Zhang and Liu (2021) in their study proposed a hybrid framework to predict the spread of COVID-19, using the impact of control measures in epidemic spreading to imbed LSTM model into a susceptible-infected-quarantined-recovered (SIQR) epidemic spreading model and optimize the parameters to obtain the optimal predictive model and control measures. The prediction results show that the proposed hybrid deep leaning has a high prediction accuracy and can be used to forecast infection and death cases to control the pandemic [15]. Verma et al., Combined Convolutional Neural network (CNN) and LSTM models to predict the daily confirmed cases of COVID-19 pandemic in four affected states of India. The prediction comparison shows that the proposed deep learning model and the stacked LSTM outperforms other trained and tested models in this study [16]. Recently, researchers have developed a lot of forecasting models to capture the complex trends and predict the infectious diseases [17]. While it is not new to predict malaria cases using imported malaria cases as the predictor and deep learning hybrid models, we attacked this problem differently. That is, studying one specific type of *Plasmodium* (that is, *Plasmodium falciparum*), which has increased in recent years with a higher fatality rate, which poses a threat to the health of labor travelers and challenges to China’s healthcare system. Additionally, it would increase the risk of malaria being reintroduced and spread to malaria-free receptive areas. We considered the effect of international traffic stagnation due to the COVID-19 pandemic.

Some of these advanced deep learning models like LSTM and GRU recurrent neural networks with a large number of discrete time steps have been used in predicting infectious diseases like malaria. However, to the best of our knowledge there’s no published study that have combined ARIMA time series forecasting model and gated recurrent unit (GRU) to predict the impact of imported malaria from African countries to the on the increase of Plasmodium falciparum cases in China before and during the COVID-19 pandemic. This article mainly studies the integration of traditional time series prediction model (ARIMA) and deep learning models (GRU) on this to achieve better prediction results. When analyzed the experimental results, it was found that using traditional times series forecasting models still has a high prediction error, which shows that the predictive ability of a single model is limited. Therefore, this study uses output value of the ARIMA model as input of the GRU model to build ARIMA-GRU hybrid model, which significantly reduced the prediction error.

## Materials and methods

Throughout this section, we discussed all the methods and materials, a description of the dataset features, the block diagram of the study, and the evaluation measures.

### Ethics statement

This study was based on official confirmed malaria cases in all 31 provinces of China. We conducted analyses at aggregate level and no confidential was involved. The research protocol was approved by the institutional review board of the Institute of Complexity Science, Qingdao University, China.

#### Data source

The number of *P*. *falciparum* cases in China from 2004 to 2016 obtained Chinese Center for Disease Control and prevention (www.phsciencedata.cn) [18]. We collected the number of imported malaria cases from African countries to China 2012–2018 from China CDC [19]. These two datasets, were used to analyze and predict the impact of imported malaria from African countries on the rise of *P*. *falciparum* cases in China before the COVID-19 pandemic.

The number of imported malaria cases from October 2020 to May 2021 in Guangdong province obtained from national information reporting system of infectious diseases [11], were used to examine the dominance of *P*. *falciparum* among other plasmodium types imported in China during the COVID-19 pandemic.

The study also analyzed the imported malaria cases during four months of the pre-and during the COVID-19 pandemic from December 1, 2019, to March 31, 2020, and the past two years of the same period (December 1, 2017, to March 31, 2018; December 1, 2018, to March 31, 2019) before COVID-19 in China by using the data obtained from The Parasitic Disease Control Information Management System.

To investigate and predict malaria deaths caused by *P*. *falciparum*, we used the number of monthly malaria deaths in China from January 1, 2011, to December 3, 2020, collected from the National Notifiable Disease Reporting System (NNDRS) [20]. The details of datasets are shown in Table 1.

**Table 1:** Details of the datasets.

Datasets	Names	Duration	Temporal resolution	Spatial resolution	Sources
1	*P*. *falciparum cases*	2004–2016	Month	31 provinces	Chinese Center for Disease Control and Prevention [17]
2	Imported malaria cases from Africa countries	2012–2018	Year	45 countries	China CDC [19]
3	Distribution of *Plasmodium* types	Oct 2020	May 2021	1 province	National information reporting system of infectious diseases [11]
4	Malaria deaths	Jan 1, 2011 to Dec 3, 2020	Month	China	Parasitic Diseases Information Reporting Management System [20]

We applied the proposed deep learning hybrid model on the monthly *P*. *falciparum* cases in all 31 provinces of China and imported malaria cases from Africa to predict the resurgence of malaria in China.

#### Statistical analysis

To investigate the impact of imported malaria from Africa on the rise of *P*. *falciparum* cases in China before the COVID-19 pandemic, we used the annual malaria cases imported from 2012 to 2018 from 45 African countries and the *P*. *falciparum* cases in 31 Provinces of China. A generalized regression model was used to examine the association of time trends between imported malaria cases from Africa and *P*. *falciparum* cases in China. Because imported malaria cases started increasing around 2012 based on the rise of business between China and African countries, a generalized regression model with multiple interaction terms of time trend was used to analyze the association with imported cases caused by both Africans entering China for different purposes, and Chinese workers returning home. To analyze the impact of COVID-19 pandemic control measures, we used three years’ interval phases: December 2017- March 2018, December 2018-March 2019, and December 2019-March 2020. We selected this period because it’s the time when Chinese people return to their home cities to celebrate the Chinese new year, the most important traditional Chinese holiday with their family. The study used statistical plots and percentage change to compare the imported cases in defined three periods.

This study used the data from October 2020 to May 2021 to analyze how imported cases from African countries affected the increase of *P*. *falciparum* cases in China during the COVID-19 pandemic.

#### Hybrid model prediction construction

To predict the *P*. *falciparum* cases and the number of malaria deaths in China before and during the COVID-19 pandemic. We built a hybrid model using monthly *P*. *falciparum* cases from 2004 to 2013 for training and validation purposes. To evaluate the performance of the hybrid model, we used the remaining falciparum cases from 2014 to 2016. Using the hybrid approach to model the *P*. *falciparum* cases, we gained a clear insight into how imported malaria cases have influenced the rise of *P*. *falciparum* cases in each province of China. There are two co-existing approaches for time series prediction, statistical and machine learning methods. Both methods come with different strengths and limitations. Hybrid methods promise to advance time series prediction by combining the best aspects of statistics and machine learning. The statistical models such as the Holt-Winters or ARIMA have been practiced for decades in modeling time series data. They stand out because they are robust and flexible. Moreover, these methods work well when few data are available and may exploit a prior knowledge.

**Fig 1:**
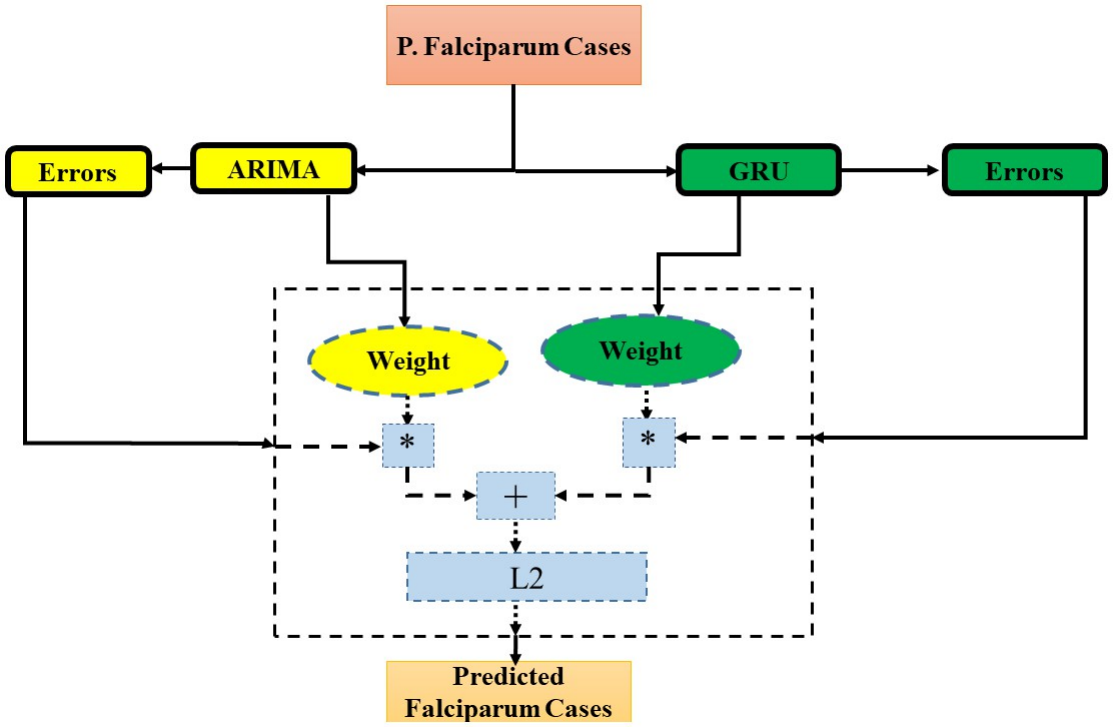
Proposed ARIMA-GRU hybrid model architecture.

Recent research has led to the successful application of machine learning methods such as Long Short-Term Memory Networks (LSTMs), Gated Recurrent Units (GRUs), or Convolutional Neural Networks (CNNs) to forecasting tasks. The advantage of these approaches is that they don’t assume linearity and have the exceptional capability of universally approximating almost any function. In addition to that, machine learning methods can exploit cross-series information to enhance an individual forecast. Besides these strengths, machine learning methods face several limitations. Apart from demanding massive amounts of data and extensive computational times, models based on machine learning have trouble extrapolating data. Although machine learning methods successfully overcome the drawback of ARIMA models in nonlinear relationships, they have produced mixed results for purely linear time series. Thus, neither ARIMA nor neural networks are solely sufficient to model a real-world time series [21]. Hybrid methods promise to advance time series forecasting by combining the strengths of statistical and machine learning methods [22]. The pipeline of the proposed model is made up of an ARIMA and a GRU models, as illustrated in Fig 1.

The fundamental idea is that this combination compensates for the limitations of one approach with the strengths of the other. For instance, the effectiveness of statistical methods with limited data availability can counteract the extensive data requirements of machine learning methods. Apart from that, taking account of a priori knowledge can simplify the expected forecasting task and decrease the computational effort. Furthermore, hybrid methods can incorporate cross-learning, something that many statistical methods lack. Finally, these approaches provide a solution to the dilemma posed by the assumption of linearity. As real-world time series may be purely linear, purely nonlinear, or often contain a combination of those two patterns, hybrid methods can be effective where traditional approaches reach their limits [23]. Based on the highlighted advantages of a hybrid model, we applied a hybrid deep learning model known as ARIMA-RNN. This study combined the ARIMA and GRU models, which perform better on sequence time series. The expression of *P*. *falciparum* cases forecasting is the sum of the linear and non-linear components. The ARIMA and GRU models evaluated linear and non-linear components, respectively. This can be mathematically depicted as:

$$P_{t}=N_{t}+L_{t+e_{t}}$$ (1)

Where *N*_*t*_ and *L*_*t*_ represent non-linear and linear components at time t, respectively. And *e*_*t*_ represents the error values of ARIMA and GRU. The ARIMA performs well on the linear problems in the data, whereas GRU perform well on the non-linear problems in the data. The forecasting equation of ARIMA is represented as ARIMA(*p*,*d*,*q*). This equation depends on lags of the dependent variable and/or lags of the forecast errors. In ARIMA(*p*,*d*,*q*), *p*, *q* respectively is the order of the Auto-Regressive (AR) model and Moving Average (MA) model, and *d* is the order of differencing applied to the data. These all values are integers. In terms of *y*, the general forecasting equation is:

$$\hat{y}=\mu +\varphi_{1y_{t-1}}+‥+\varphi_{py_{t-p}}-\theta_{1}e_{t-1}-‥-\theta_{qe_{t-q}}$$ (2)

The term *μ* is a constant; *φ*_*k*_ and *θ*_*k*_ are the value of the coefficients of the AR model variable *y*_*t-k*_, and MA model variable *e*_*t-1*_. The signs of the moving average parameters *θ* are negative, following the convention introduced by Box and Jenkins. We have used the Alkaline Information Criterion(AIC) for the parameter estimation step.


$$AIC=-2\;ln\left(\hat{L}\right)+2K$$ (3)

The $ln(\hat{L})$ notation is the value of the likelihood function, and *k* is the degree of freedom, that is, the number of parameters used. A model with a small AIC value is generally considered a better model. There are different ways to compute the likelihood function, $ln(\hat{L})$. We used the maximum likelihood estimator for the computation. This method tends to be slow but produces accurate results. As a final step, we use the residual from the ARIMA model as input to the GRU model. Since the ARIMA model has defined a linear pattern, the residual should encompass non-linear characteristics.

The LSTM and GRU models have performed well in capturing long-term dependencies. The gated recurrent unit is the focus of the study. GRU is an improved recurrent neural network as a simple variant of LSTM by combining the input gate and forgetting gate into a single gate called update gate. GRU comprises the update and reset gates. The update gate decides how much information to save from the previous time steps. The reset gate determines which information to discard from the preceding time step, and it can only control information inside the unit because it has no additional memory cell. The hidden state *ht* of the GRU at time *t* is a linear interpolation between the prior state *ht* − 1 and the candidate state $\tilde{h}$ such that:

$$h_{t}=\left(1-z_{t}\right)h_{t-1}+z_{t}\tilde{h}$$ (4)

in which an update gated *Z*_*t*_ determines how much the unit will update the activation. The update gate is computed as:

$$z_{t}=\sigma \left(W_{z}x_{t}+U_{z}h_{t-1}\right)$$ (5)

where *W*_*z*_ is the weight matrix that connects the input vector *x* at time *t* to the hidden layer neurons, *U*_*z*_ is the weight matrix that connects hidden layer neurons to the neurons at time *t*-1 and *t* and *σ* is the logistic sigmoid function such that:

$$\sigma \left(X\right)=\frac{e^{x}}{1+e^{x}}$$ (6)

And the candidate state is computed as:

$${\tilde{h}}_{t}=tanh(Wx_{t}+U(r_{t}h_{t-1}))$$ (7)

Where *tanh* is the hyperbolic tangent function such that:

$$tanh\left(x\right)=\frac{e^{x}-e^{-x}}{e^{x}+e^{-x}}$$ (8)

When *r*_*t*_ is close to 0, the reset gate effectively makes the unit read as if it was the first symbol in the sequence, which allows it to forget the data from the prior unit. The reset gate *r*_*t*_ is computed as:

$$r_{t}=\sigma (x_{t}W_{r}+U_{r}h_{t-1})$$ (9)

We generate predictions for each 2-time step to calculate the residual value after fitting the ARIMA model. Then each data’s last data point will act as the target variable Y and the remainder as the variable X. For the next GRU model, the new X/Y-split datasets will be the input values. For the GRU model, the residual values derived from the ARIMA model were used as input. In this study, the model architecture is an RNN that employs 125 units of GRU. The final outputs of GRU units are combined into a single, fully connected layer value. Then the value would be transferred to produce a single final prediction via a doubled hyperbolic tangent activation function. Apart from dropout, L2 regularization, were employed to avoid overfitting by preventing the weights of each network from being too high in the GRU model. Each layer’s high parameter values can cause the network to concentrate severely on a few features, which can lead to over-fitting.

#### Model validation

We evaluated the performance of the proposed ensemble model and its constituents using R^2^ scores, mean absolute error (MAE), and root mean squared error (RMSE) loss functions.

The RMSE evaluates the continuous variables by measuring the average differences between predicted and observed error values. R^2^ measures how well the regression line replicates the actual outcomes. It gives a clear insight into the adjustment between variance and independent variable in the model and implicates its greatness for the dataset. Mathematically they are depicted as follows:

$$R^{2}=1-\frac{\sum {\left(y_{t}-\bar{y}\right)}^{2}}{\sum {\left(yt-\hat{y}\right)}^{2}}$$ (10)

$$MAE=\frac{1}{N}\sum_{t=1}^{N}\left|y_{t}-{\hat{\text{y}}}_{t}\right|$$ (11)

$$RMSE=\sqrt{\frac{1}{N}\sum_{t=1}^{N}{\left(y_{t}-{\hat{\text{y}}}_{t}\right)}^{2}}$$ (12)

$$MSE=\frac{1}{N}\sum_{t=1}^{N}{\left(y_{t}-{\hat{\text{y}}}_{t}\right)}^{2}$$ (13)

where *N* is the number of data points, *y* is the actual output of imported malaria cases at time *t*, *ŷ* is the predicted output of the imported cases. R^2^ score tells how well the performed and strong predictor variables are related to the target variable of the trained model. MAE calculates the absolute difference between the actual output and the predicted value of imported malaria cases.

## Analysis and simulation results

This section analyses imported malaria from African countries versus the increase of *P*. *falciparum* in China before and during the COVID-19 pandemic. It also analyzes the performance of the proposed prediction hybrid model compared to its constituent models on the *P*. *falciparum* cases and the number of deaths in China caused by *P*. *falciparum* cases. A total of 20,938 imported malaria cases from 67 countries were identified between 2012 and 2018 in China. Among them, 64.5% were *P*. *falciparum* cases. The proportion of imported malaria cases increased from 2012 (n = 2,474, 91.0%) to 2018 (n = 2,5511, 99.7%). The number and proportion of imported *P*. *falciparum* cases increased from 2012 (n = 1403, 57.4%) to 2018 (n = 1655, 66.0%). Among the total imported malaria cases, 16,720 cases (79.9%) were from African countries. These cases imported from African countries showed an increasing trend of proportion from 2012 (n = 1,454, 58.8%) to 2018 (n = 2,470, 90.4%).

**Fig 2:**
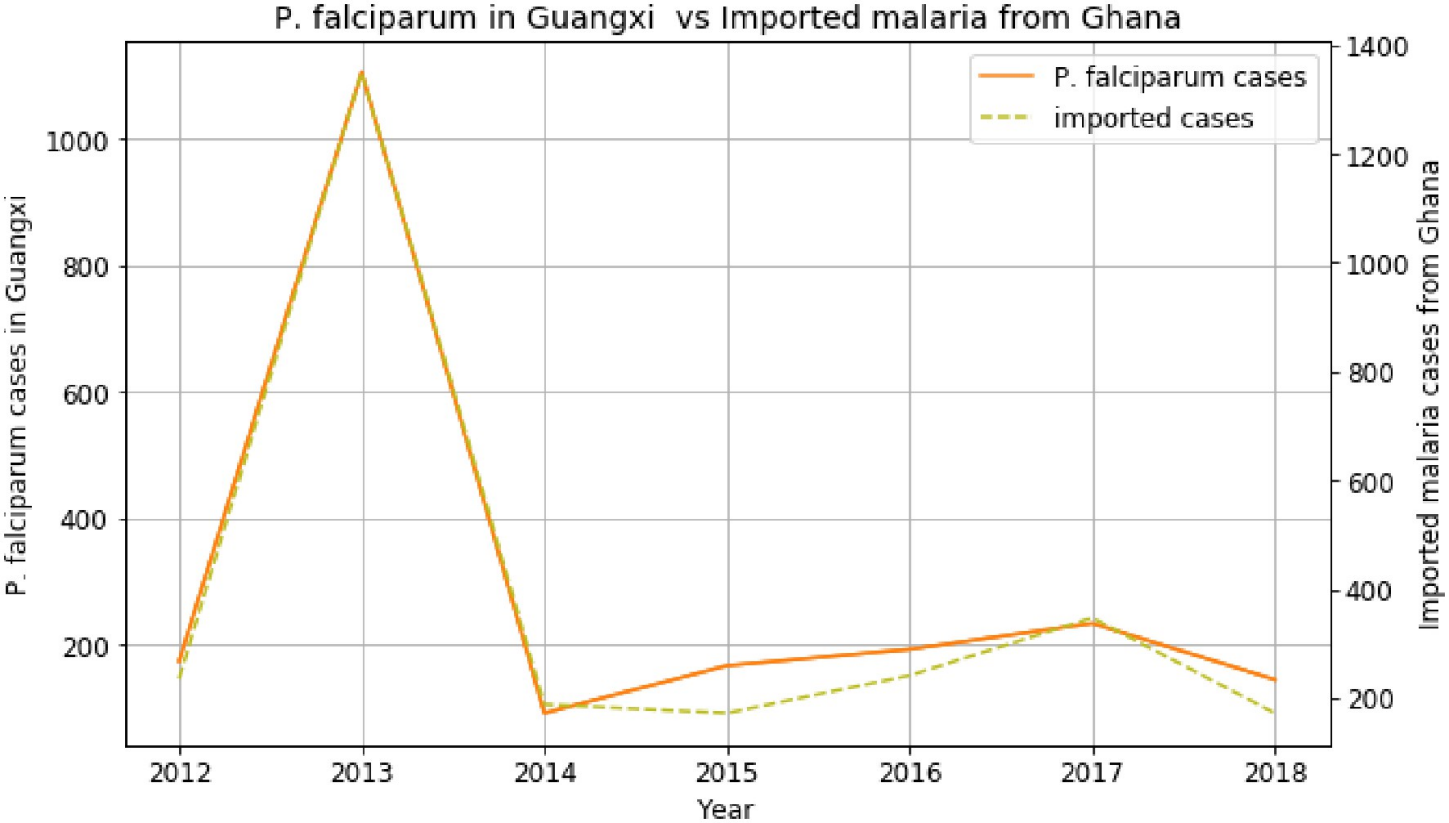
Association between *P*. *falciparum* cases in Guangxi and imported cases from Ghana before COVID-19.

Firstly, we analyze the impact of imported malaria cases from 45 African countries on the increase of *P*. *falciparum* cases in different provinces of China. As shown in Fig 2 for Ghana and Guangxi province, we can see that *P*. *falciparum* cases in Guangxi increased along with imported malaria cases from Ghana in 2013. As mentioned in the introduction section, many Chinese gold miners’ workers returned to Guangxi from Ghana in 2013. Several returning workers in 2013 were malaria-infected, explaining the peak of imported malaria cases. Furthermore, its importation from Ghana demonstrates its impact on *P*. *falciparum* cases in Guangxi [9].

**Fig 3:**
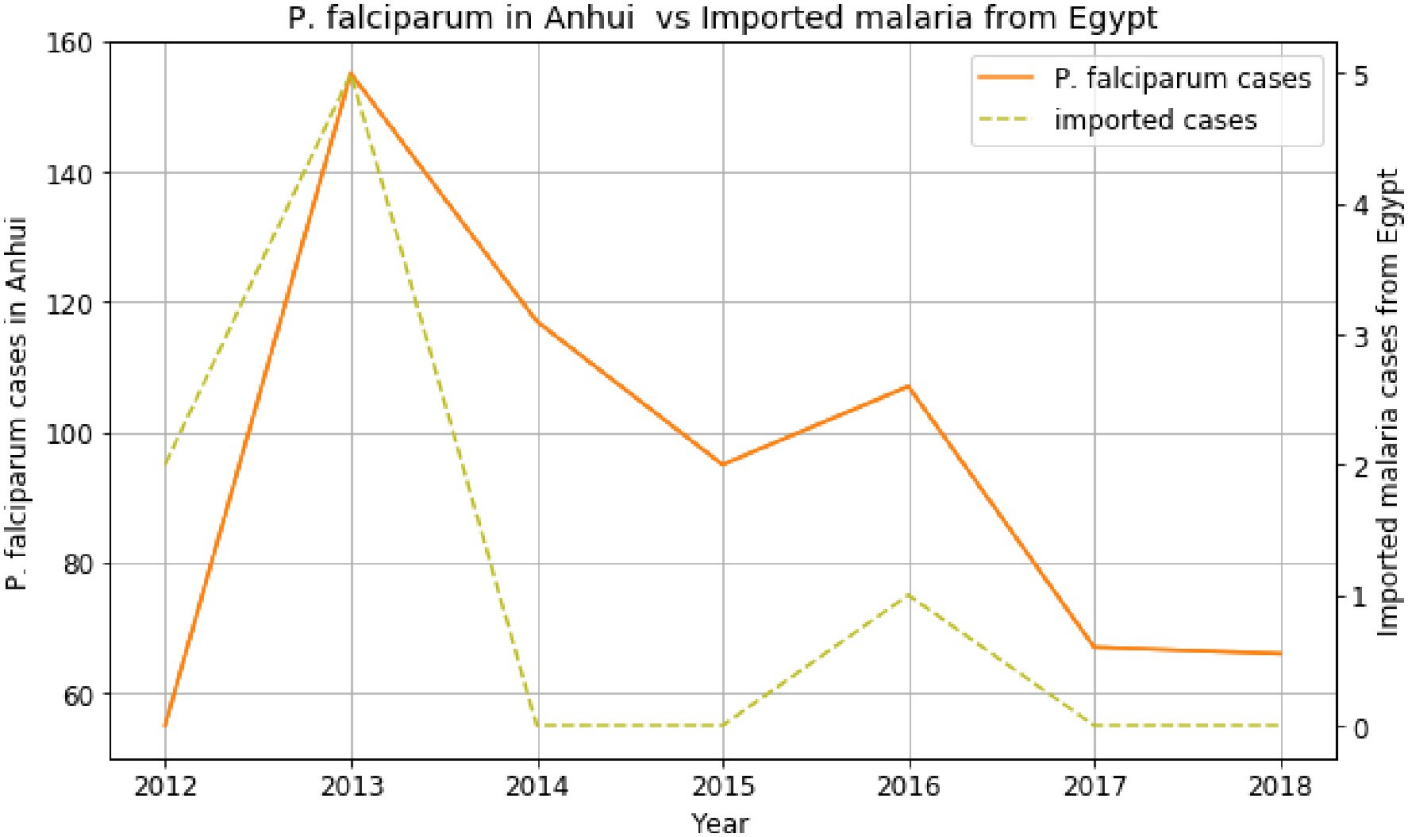
Association between *P*. *falciparum* cases in Anhui and imported cases from Egypt before COVID-19.

Fig 3 shows the direct effect of the fewer cases from Egypt to *P*. *falciparum* in Anhui province, which trended along in 2013 and 2016.

**Fig 4:**
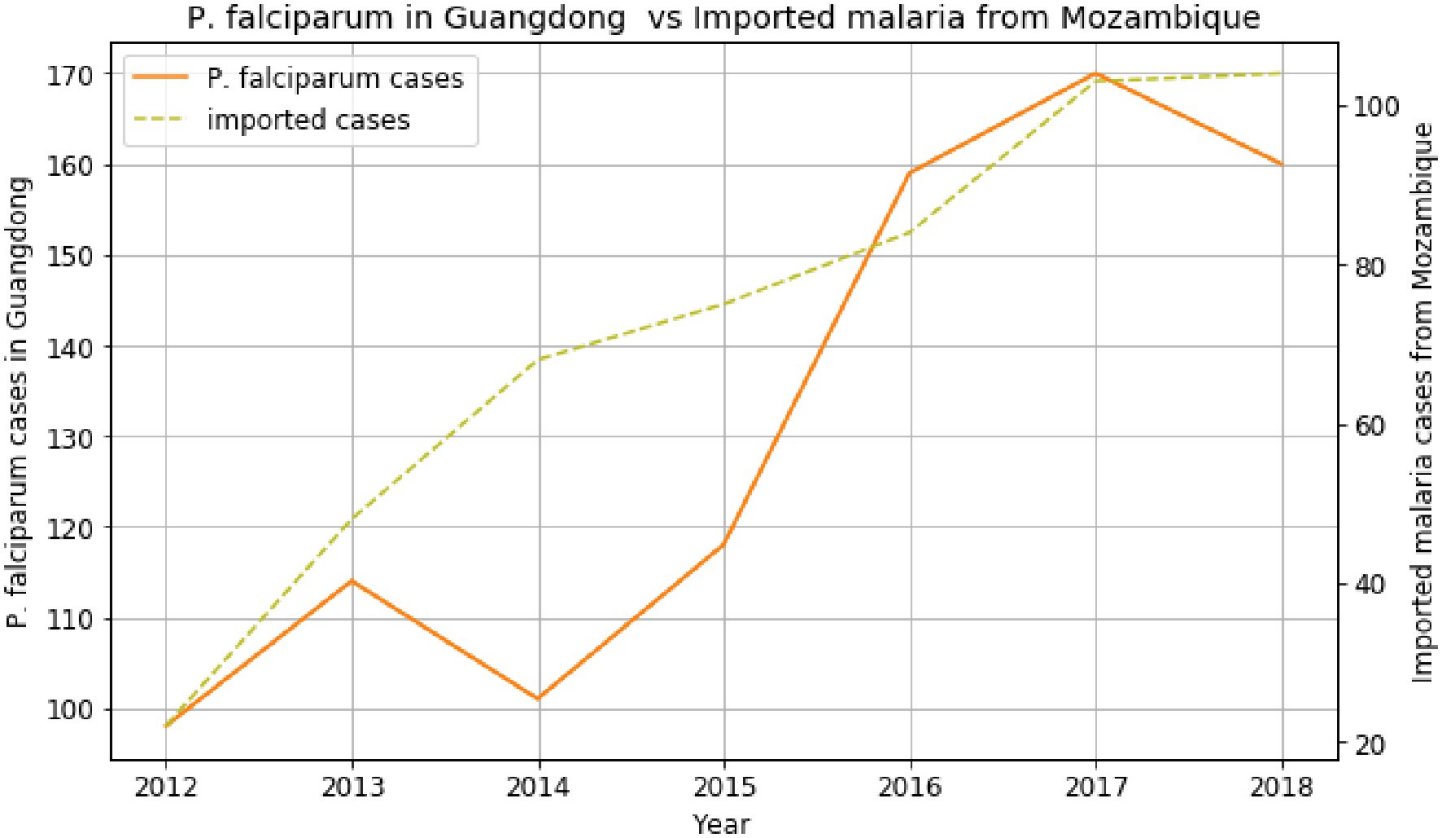
*P*. *falciparum* cases in Guangdong versus imported cases from Mozambique before COVID-19.

Fig 4 shows the rise of imported malaria cases from Mozambique from 2012 to 2017. This rise influences the increase of *P*. *falciparum* cases in Guangdong province except for 2013–2014. The two plots peaked in 2017, showing the direct effect of imported cases from Mozambique on the *P*. *falciparum* cases in Guangdong province [24].

**Fig 5:**
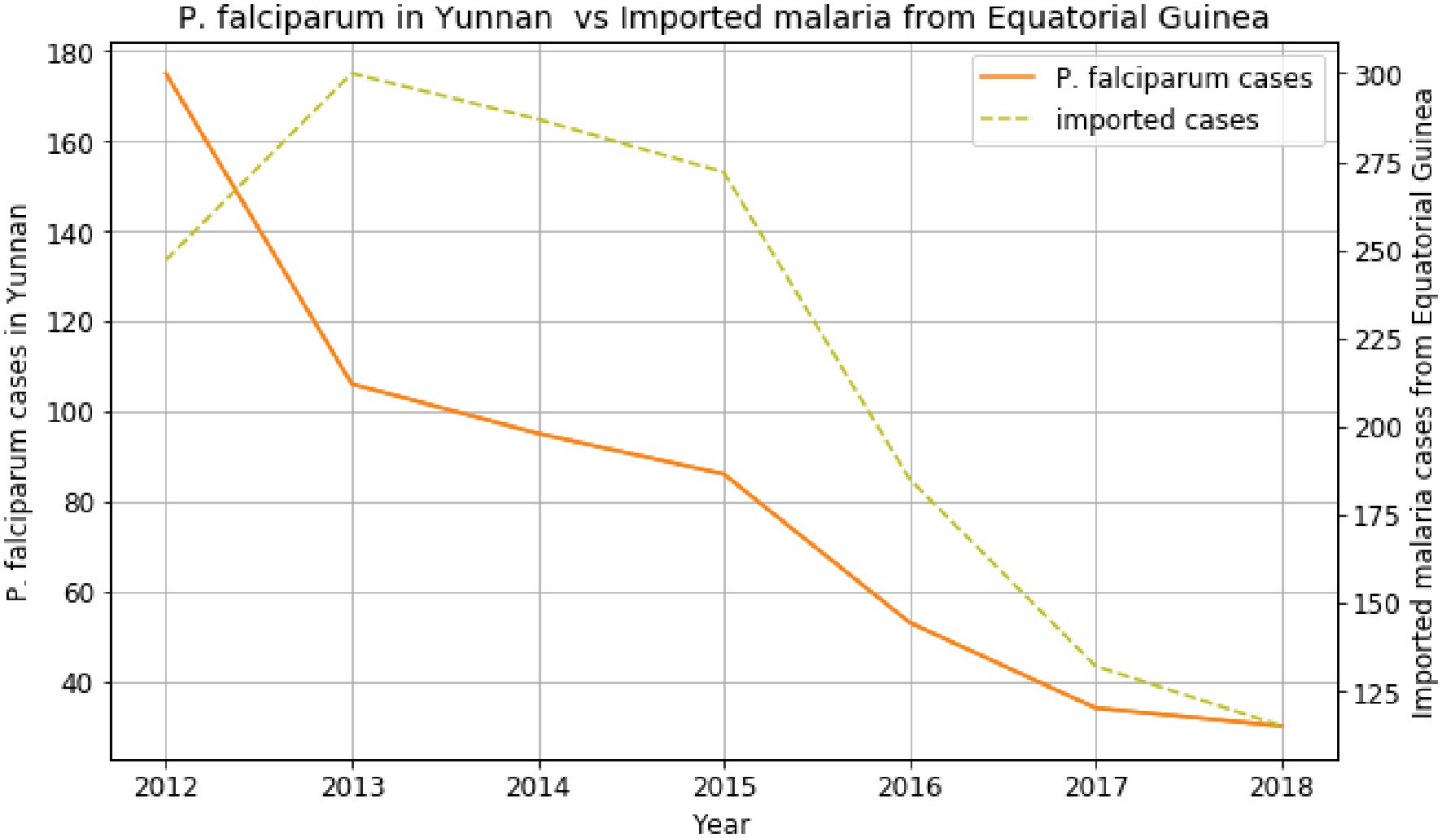
*P*. *falciparum* cases in Yunnan versus imported cases from Eq. Guinea.

Contrary to Fig 4 above, Fig 5 shows the decline of imported malaria cases from Equatorial Guinea with *P*. *falciparum* cases in Yunnan province. We can notice an increase in imported malaria from Equatorial Guinea, which directly affected the slight rise of Yunnan *P*. *falciparum* cases in 2015. The trends continued declining slightly and hit a trough in 2018 but *P*. *vivax* increased in 2019 in Yunnan [25].

The trend analysis of *P*. *falciparum* cases in many Chinese provinces showed similar trends to the imported malaria cases from many African countries. The imported cases were mainly from Ghana (n = 2,704, 12.9%), Angola (n = 2,085, 10.0%), and Nigeria (n = 1,939, 9.3%).

**Fig 6:**
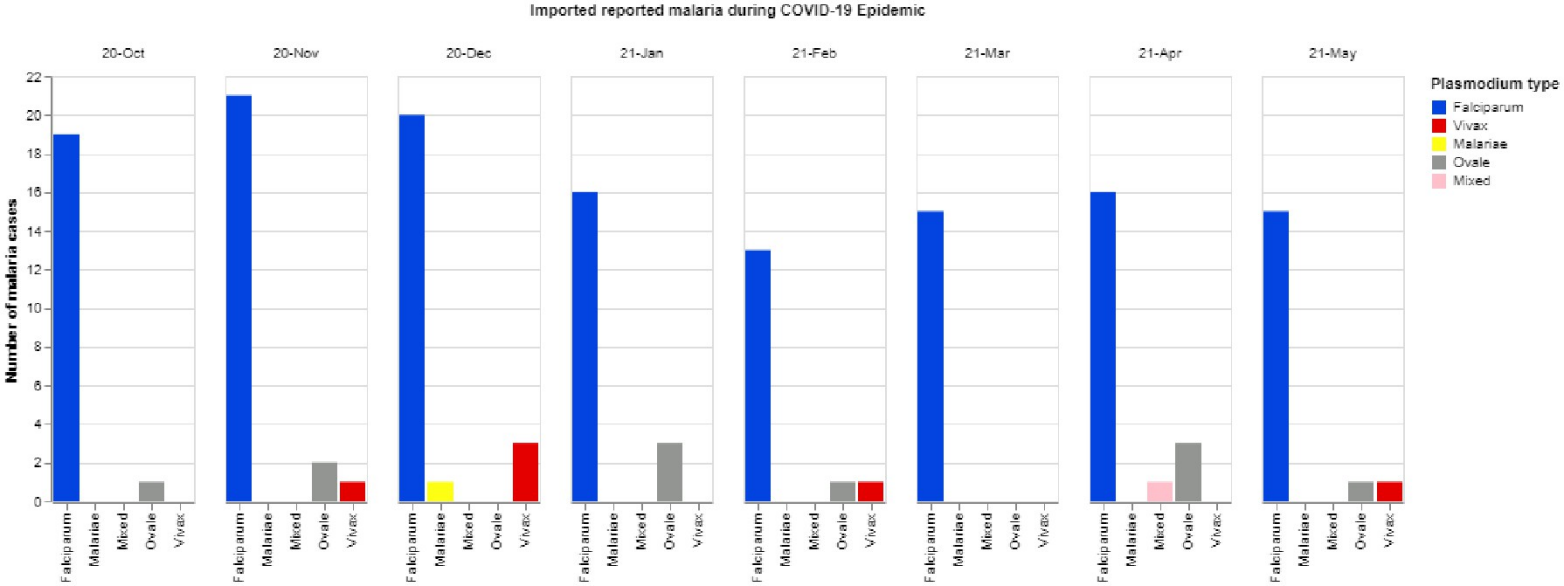
The dominance of *P*. *falciparum* cases imported in Guangdong during COVID-19.

Secondly, we analyze the intervention of COVID-19 pandemic control measures that have affected *P*. *falciparum* cases in Guangdong provinces based on imported malaria cases from African countries. Fig 6 shows the predominant of the *P*. *falciparum* cases compared to other *Plasmodium* species in Guangdong province during the COVID-19 pandemic. Our analysis used the data collected based on key flights and populations from malaria-affected areas in Guangdong province from October 2020 to May 2021. Entry personnel from malaria-affected areas were subject to surveillance for malaria. There were at least 100,000 tests conducted in the Guangdong Province; 154 confirmed malaria cases occurred during this surveillance.

The results identified *P*. *falciparum* as a high percentage (87.7%; 135/154 of the cases) among other species. During the COVID-19 pandemic, the imported malaria cases in Guangdong province were from 23 African countries, including Nigeria, Cameroon, Congo, Uganda, and the Democratic Republic of Congo (DRC) as the top 5 countries with the highest number of imported malaria, with a combined percentage of 61.7% of the total cases. The infected patients were the residents of 22 provinces, of which Guangdong, Hunan, Henan, Shandong, and Hubei as the top 5 provinces of residence with 51% of all the identified cases. Among these imported malaria cases during the COVID-19 pandemic, there was one death caused by the *P*. *falciparum* case.

As analyzed on the imported malaria cases from African countries from 2012 to 2018 before COVID-19, still the same countries have a high number of imported cases from Africa, and most of the provinces with a large number of *P*. *falciparum* cases in China appeared on the top of the patients’ residents during the COVID-19 pandemic. Despite the decreased number of imported malaria cases in China due to the stagnation of the international traffic during the COVID-19 pandemic, *P*. *falciparum* cases have managed to stay on top of other *Plasmodium* species and African countries with the highest imported malaria cases.

Thirdly, we analyzed the number of malaria deaths caused by imported malaria cases with the highest proportion of *P*. *falciparum* before and during the COVID-19 pandemic at the national level. The National Notifiable Disease Reporting System (NNDRS) recorded 164 malaria deaths in China caused by imported malaria cases from January 2011 to December 2020 [20]. Among the recorded malaria deaths, 97% were due to the *P*. *falciparum cases* imported from African countries. The number of malaria deaths decreased from 2011 (n = 30) to 2020 (n = 6), a result statistically evaluated by the trend chi-squared test, χ2 = 322.153, P<0.001. Since the COVID-19 pandemic was identified in Wuhan, China, from December 2019 to December 2020, 9 malaria deaths were recorded in China.

**Fig 7:**
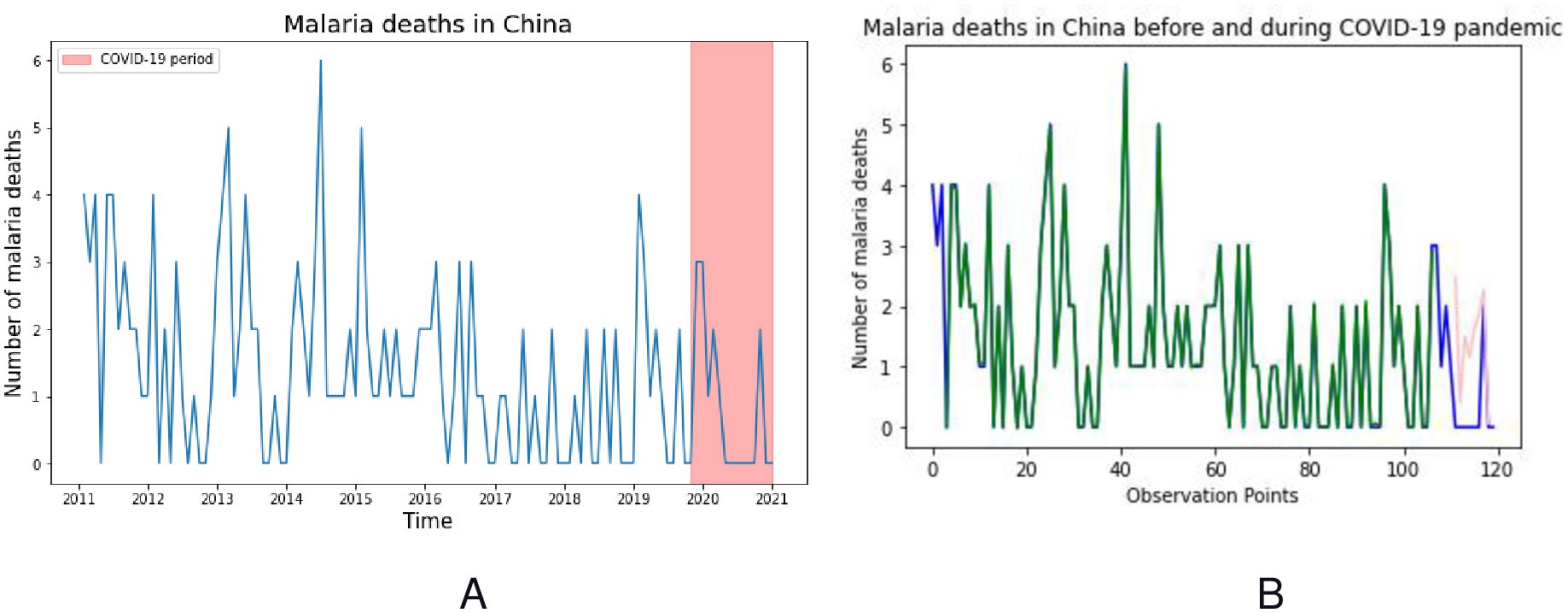
(A) Time series of malaria deaths in China before and during COVID-19 pandemic, (B) ARIMA-GRU prediction of malaria deaths, the blue line depicts the observation data, the green line depicts the training data and red line depicts the test predicted.

The analysis showed that the increase of imported *P*. *falciparum* cases and malaria deaths occurred in January and February when Chinese laborers returned to their home cities from African countries to celebrate the Chinese spring festival. The delay in diagnosis and treatment of the imported *P*. *falciparum* cases are the major causes of malaria deaths in China before and during the COVID-19 pandemic. Using the ARIMA-GRU hybrid proposed approach, we analyzed and predicted the time series of malaria deaths from January 2011 to December 2020, as presented in Fig 7. The time series of malaria deaths in China (A) in Fig 7, shows the seasonality of malaria deaths in blue line with the highlighted part in red during the COVID-19 pandemic. For the prediction part B of the figure, the proposed model performed better than its constituent alone, as shown in Table 2 below.

**Table 2:** Comparing the performance of the proposed hybrid and the base models on the malaria deaths in China before and during COVID-19 Pandemic.

	*ARIMA*	*GRU*	*ARIMA-GRU*	*LSTM*	*ARIMA-LSTM*
*RMSE*	0.109	0.121	0.017	0.124	0.056
*MAE*	0.036	0.059	0.013	0.087	0.092

**Fig 8:**
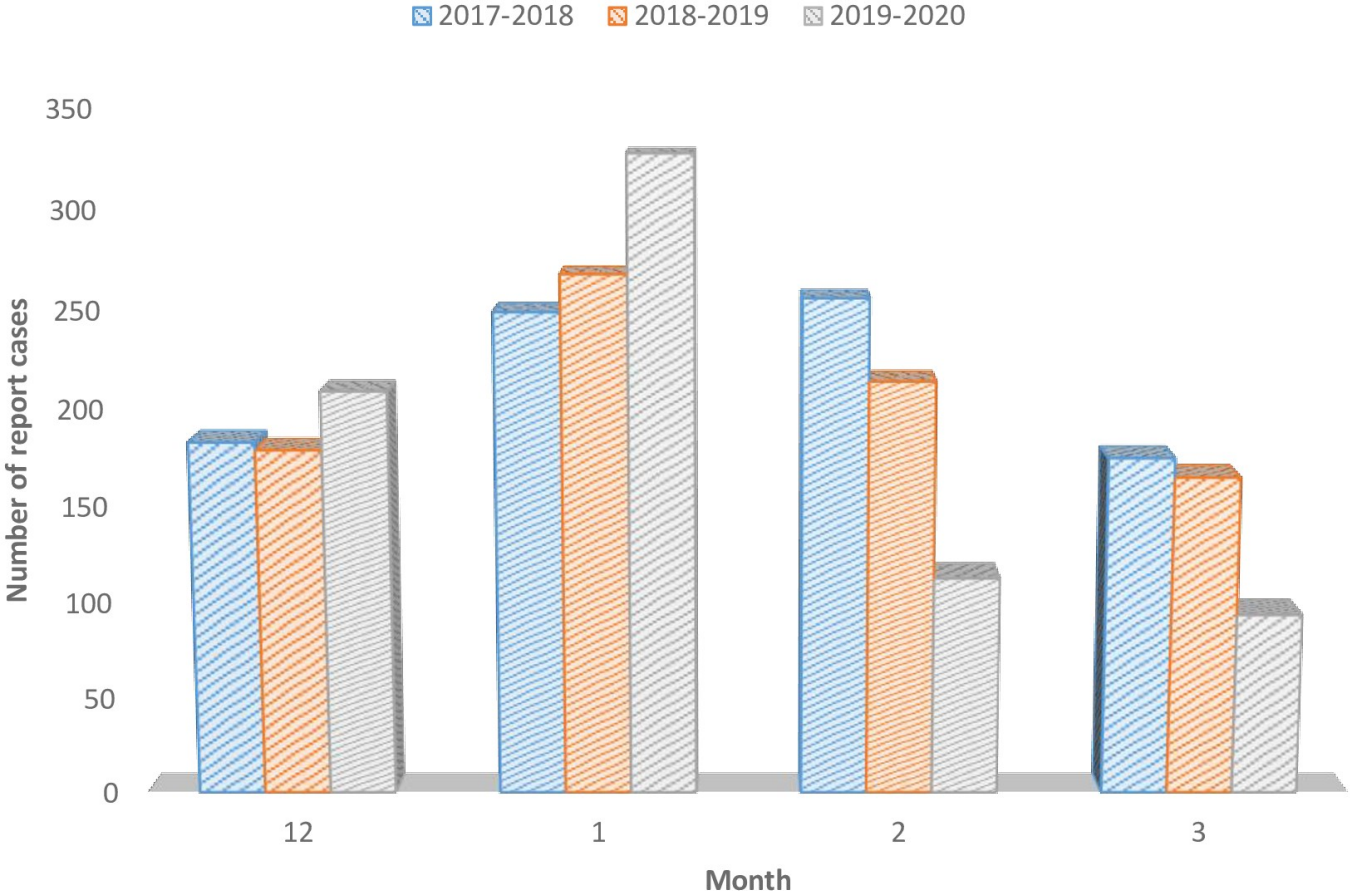
Comparison of the imported malaria cases between pre- and during COVID-19.

For the fourth part of the analysis of the results, as can be seen in Fig 8 above, we investigated the situation of imported malaria cases and *P*. *falciparum* cases at a specified time before and during the COVID-19 pandemic, international traffic control measures at the beginning.

We analyzed the imported malaria cases during four months of the pre-and during the COVID-19 pandemic from December 1, 2019, to March 31, 2020, and the past two years of the same period (December 1, 2017, to March 31, 2018; December 1, 2018, to March 31, 2019) before COVID-19 in China. A total of 863 imported malaria cases were reported from December 2017 to March 2018, which decreased to 826 malaria cases from December 2018 to March 2019 and finally, from December 2019 to March 2020, with 750 imported malaria cases in China. The number and proportion of imported malaria cases increased from African countries in the first period (n = 779, 90.3%) to 787 (95%) of the total imported cases in the second period (2018–2019), then the proportion decreased a little bit in 2019–2020 (706, 94.1%). The highest number of imported malaria cases occurred in January due to the return of Chinese people to celebrate the Chinese new year in their home provinces. In all three periods defined, African countries have been the predominant origins of imported malaria cases in China, which caused the increase of *P*. *falciparum* cases in different provinces of China.

***P*. *falciparum* cases prediction by ARIMA-GRU hybrid model**. The analysis showed an emerging link between the rise of imported malaria cases from Africa and *P*. *falciparum* cases in many provinces of China. Many imported malaria cases from Africa were *P*. *falciparum* cases. Based on these factors, we proposed a statistical and deep learning hybrid approach to model the resurgence of malaria in China using monthly data of *P*. *falciparum* from 2004 to 2016.

Using the performance evaluation measures defined in the materials and methods section of the study, Table 3 shows the comparative performance between the proposed hybrid model, GRU, LSTM and ARIMA-LSTM model on *P*. *falciparum* cases monthly data from 2004 to 2016 in all provinces of China. The proposed model achieved small prediction errors in most of the provinces of China.

**Table 3:** Comparing the performance of the proposed hybrid model and GRU model on *P*. *Falciparum* cases before the COVID-19 pandemic.

Province	GRU		ARIMA-GRU		LSTM	ARIMA-LSTM
	RMSE	MAE	RMSE	MAE	RMSE	MAE
**ANHUI**	0.18	0.15	0.09	0.06	0.3564	0.1873
**BEIJING**	1.19	0.96	0.56	0.70	0.1705	0.0342
**CHONGQING**	0.96	0.80	1.03	0.78	0.3939	0.1881
**FUJIAN**	2.03	1.64	1.83	1.37	0.7635	0.2016
**GANSU**	1.04	0.84	0.90	0.83	0.7464	0.2712
**GUANGDONG**	1.62	1.34	1.3	1.15	0.6247	0.3091
**GUANGXI**	0.40	0.23	0.32	0.11	0.5329	0.3249
**GUIZHOU**	1.08	0.13	0.09	0.11	0.7133	0.6098
**HAINAN**	0.32	0.15	0.10	0.08	0.5438	0.3222
**HEBEI**	1.23	0.91	0.90	0.38	0.6683	0.3117
**HEILONGJIANG**	0.81	0.59	0.31	0.22	0.6242	0.5628
**HENAN**	3.34	2.05	1.20	0.12	0.6533	0.5573
**HUBEI**	2.92	1.51	2.23	1.377	0.5277	0.3252
**HUNAN**	2.925	1.942	2.755	1.77	0.37669	0.1827
**INNERMONGOLIA**	0.28	0.15	0.20	0.09	0.0596	0.0361
**JIANGSU**	4.25	2.85	2.89	2.42	1.9506	1.2374
**JIANGXI**	1.10	0.77	1.15	0.03	0.6352	0.4357
**JILIN**	0.61	0.43	0.10	0.17	0.6185	0.4558
**LIAONING**	1.11	0.67	0.62	0.48	0.1213	0.0224
**NINGXIA**	0.15	0.13	0.01	0.07	0.1579	0.1530
**QINGHAI**	0.78	0.19	0.16	0.06	0.1829	0.0554
**SHAANXI**	0.15	0.09	0.15	0.07	0.8312	0.6778
**SHANDONG**	1.79	1.28	1.03	1.23	0.6412	0.4879
**SHANGHAI**	1.09	0.83	0.95	0.81	0.5056	0.2166
**SHANXI**	0.15	0.09	0.14	0.07	0.1539	0.0626
**SICHUAN**	4.21	3.01	4.26	3.03	0.5023	0.3693
**TIANJIN**	0.63	0.51	0.31	0.12	0.3087	0.1504
**TIBET**	0.09	0.15	0.71	0.001	0.1017	0.0177
**XINJIANG**	0.28	0.10	0.30	0.22	0.2872	0.1367
**YUNNAN**	0.59	0.52	0.44	0.34	0.6099	0.3743
**ZHEJIANG**	2.56	1.99	2.48	1.81	0.4404	0.1768

**Fig 9:**
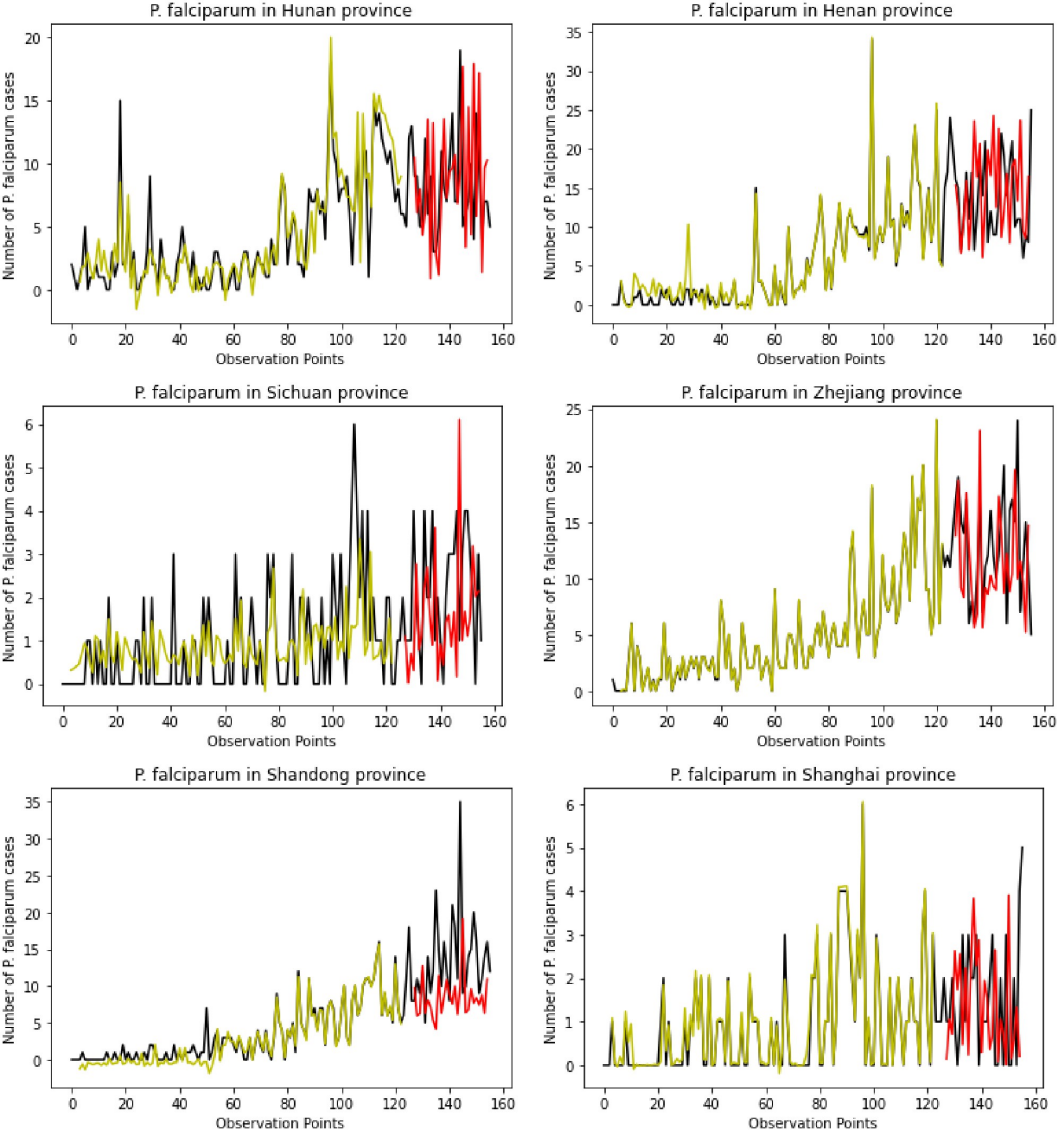
Observed monthly *P*. *Falciparum* cases (black lines) and ARIMA-GRU model predicted cases (yellow lines for training cases and red lines for testing cases).

**Fig 10:**
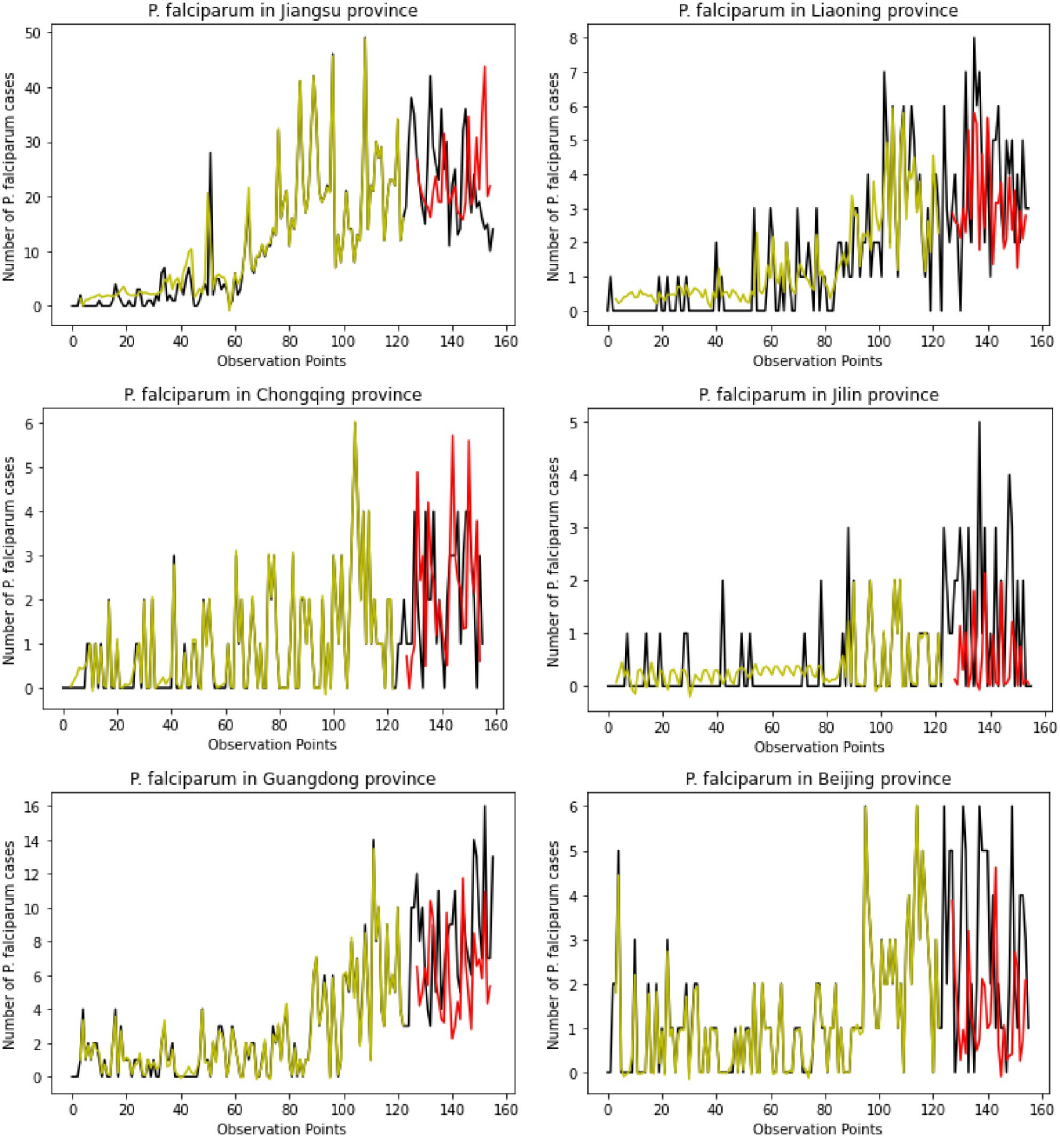
Monthly *P*. *Falciparum* cases and ARIMA-GRU model predicted cases.

Figs 9 and 10 presents the prediction curves of 12 provinces that showed the increasing trends of *P*. *falciparum* cases, such as Guangdong, Zhejiang, Hunan, Henan, Sichuan, Shanghai, and Shandong, Jiangsu, Liaoning, Chongqing, Jilin, and Beijing. The prediction figures obtained using the proposed ARIMA-GRU hybrid model showed the strength of combining statistical and deep learning models by achieving a high average prediction accuracy on the testing dataset of 96%. In analyzing the impact of COVID-19 pandemic control measures from October 2020 to May 2021 dataset, among the 22 provinces with a high number of *P*. *falciparum-infected* people during COVID-19, Guangdong, Hunan, Henan, and Shandong were the highlighted provinces with an increasing trend of *P*. *falciparum* cases. Most of these provinces are in the predicted results in the figures below with a high prediction scores using the proposed approach showing a possibility of an increase in the future due to the imported malaria cases from African countries.

## Discussion

This study examined the impact of imported malaria cases from African countries on the rise of *P*. *falciparum* cases in 31 provinces of China before and during the COVID-19 pandemic. The study also analyzed and predicted the number of malaria deaths caused by the imported *P*. *falciparum* cases from African countries before and during the COVID-19 pandemic, using the ARIMA-GRU hybrid approach, which is a combination of statistical and deep learning models. To have a clear picture of imported malaria cases from African countries and their impact on *P*. *falciparum* in China during the COVID-19 pandemic. We used the data collected in Guangdong provinces based on the key flights and populations from October 2021 to May 2021. Finally, we predicted the monthly *P*. *falciparum* cases in 31 provinces of China using the proposed hybrid model.

To analyze the impact of imported malaria cases from African countries on the rise of *P*. *falciparum* cases in China. This study focused on the seven years from 2012 to 2018, as imported malaria cases from Africa increased around 2012 [19]. The trends and generalized regression model showed an impressive association and impact between the imported cases from African countries and *P*. *falciparum* cases in China.

The trends of imported cases from African countries like Ghana, Mozambique, Equatorial Guinea, and Egypt are almost similar to the *P*. *falciparum* cases in the provinces of China, such as Guangxi, Guangdong, Yunnan, and Anhui. It explains the increase of *P*. *falciparum* in many regions of China. Based on the data and analyses presented here, we can identify an emerging route of *P*. *falciparum* transmission from Africa to China within the last two decades. The first route is the imported malaria cases from Ghana, Gabon, Benin, South Africa, and Angola, which contributed to the increase of *P*. *falciparum* cases in Guangxi province from 2012 to 2018. The imported malaria cases from DRC, Nigeria, Cameroon, Angola, Equatorial Guinea, and Congo have influenced the increase of *P*. *falciparum* cases in Guangdong province. There are many origins and destination routes identified in this study, where imported malaria from many African countries affected the rise of *P*. *falciparum* cases in the provinces of China [5].

For the analysis of COVID-19 control measures intervention on the effect of imported malaria cases from African countries to the increase of *P*. *falciparum* cases in China, the study explored monthly data from October 2020 to May 2021 in Guangdong. Guangdong province has historically been one of the most affected provinces in China by malaria. The most predominant strains were *Plasmodium falciparum* before and during the COVID-19 pandemic. The analysis showed that *P*. *falciparum* has a very high percentage compared to other imported *Plasmodium* species identified in Guangdong province.

The bar plots in Fig 6 show that *P*. *falciparum* cases from October 2020 to December 2020 are high compared to the remaining months of 2021, which happened due to the stagnation of international traffic during the COVID-19 pandemic. Many countries limited international travel and implemented border control as a global response to the crisis.

Traveling from African countries to China before the COVID-19 pandemic has been cited as a major contributor to the increase of imported malaria cases in China. Since 2010, autochthonous malaria was eliminated in most of the provinces of China, and only imported cases started to increase two years later, as in the first part of the analysis. The number of imported malaria cases reduced remarkably during the COVID-19 pandemic, as presented in Fig 6, even though *P*. *falciparum* cases were still predominant during the whole study period in China.

In predicting the number of malaria deaths in China before and during the COVID-19 caused by the imported *P*. *falciparum* cases from African countries, the study used the monthly data from January 2011 to December 2020. The ARIMA-GRU hybrid proposed approach achieved a remarkable result in predicting the number of malaria deaths at the national level compared to its constituents and previous published studies [26]. The analysis showed that among 164 malaria deaths recorded in China from January 2011 to December 2020, only nine malaria deaths occurred since the COVID-19 was identified in Wuhan, China, from December 2019 to December 2020. As presented in Fig 7 on the prediction of malaria deaths in China, malaria deaths caused by the imported *P*. *falciparum* cases from African countries decreased during the COVID-19 pandemic.

In further investigation, the study analyzed and compared the number of imported malaria in the same months two years before the COVID-19 pandemic with the number of imported malaria during the pandemic in the same period (December 2017-March 2018, December 2018-March 2019, and December 2019 to March 2020) at a national level. The analysis showed that imported malaria cases decreased in December 2019, increased at the beginning of the COVID-19, and significantly decreased in March 2020 from 185 to 94 imported malaria cases due to the COVID-19 pandemic border-crossing control measures, that decrease in imported malaria cases occurred in February and March 2020. The decline in the imported malaria cases also affects the fall of *P*. *falciparum* cases in China during the COVID-19 pandemic.

Finally, the last objective of this study was to predict the monthly *P*. *falciparum* cases from January 2004 to December 2016 before the COVID-19 pandemic. This prediction and the analyses made through our study help analyze the possibility of malaria resurgence in China due to the rise of *P*. *falciparum* cases caused by the increase of imported malaria cases from African countries. The study proposed the ARIMA-GRU hybrid approach to train the dataset and evaluate its performance using the testing dataset. The proposed hybrid model outperformed the GRU model with a high average prediction score on the monthly *P*. *falciparum* cases in all the provinces of China. Fig 9 shows the curves of 12 provinces with an increasing prediction trend which also appeared among the 22 provinces with a high number of imported *P*. *falciparum* cases during the COVID-19 pandemic from October 2020 to May 2021.

This study showed that the number of imported malaria cases from African countries was strongly associated with the number of *P*. *falciparum* cases in many provinces of China and is the deadliest among the four main types of human malaria [27]. The study shows that imported malaria cases from African countries have remarkably decreased during the COVID-19 pandemic due to the stagnation of international traffic [28]. However, *P*. *falciparum cases* from African countries may increase after the normalization of international traffic due to the association between imported malaria cases and international traffic. Thus imported *P*. *falciparum* cases from African countries are a new challenge for malaria re-introduction in China. Despite the remarkable progress made in reducing the global malaria burden, the disease remains endemic in many regions of Africa, and adequate traveler prevention measures are still insufficient. This study shows a remarkable performance of the ARIMA-GRU approach in modeling small and not complex datasets, which is hard to achieve when using only deep neural network models.

### Limitations of the study

Despite the remarkable performance of the ARIMA-GRU approach, there are some limitations in our study. We did not consider the effect of other factors such as climatic factors because this work only focuses on non-climatic factors. We will use the climatic factors in our future research, we believe another comparison using climatic and non-climatic factors would be a good follow-up research focus.

The iterative process of training gated recurrent unit converges fast, because of the randomness of the data used for gated recurrent modeling is reduced after the fitting process of ARIMA modeling. This may lead to overfitting of the proposed hybrid model in some cases. In our future work, we will scale the input data and add more data.

We could not obtain accurate predictions in some provinces by using the hybrid proposed model, due to the lack other relevant potential factors that might contribute to the rise of Plasmodium falciparum in China. In our future study, we should increase our dataset and consider other factors such weather, economic and measure factors that are in place to reduce the number of imported malaria cases in China. We will also explorer more advanced deep learning models to improve the prediction accuracy.

## Conclusion

Imported malaria is still a public health issue in countries like China that have eliminated malaria, as it can cause the resurgence of malaria. This study provided the analysis and prediction that the increase of imported malaria cases from African countries has affected the rise of *P*. *falciparum* cases in China before the COVID-19 pandemic. The study provided an analysis of the reduction of *P*. *falciparum cases* and deaths caused by imported *P*. *falciparum* cases during the COVID-19 pandemic due to the control measures regarding the limitation of international travel in China. The Chinese government has to prepare the imported malaria control measures after the normalization of international travel, to prevent the resurgence of malaria disease in China.

## References

[1] Checkley Anna M., et al. "Risk factors for mortality from imported *falciparum* malaria in the United Kingdom over 20 years: an observational study." *Bmj* 344 (2012). 10.1136/bmj.e2116 PMC3314185 22454091

[2] Askling Helena H., et al. "Management of imported malaria in Europe." *Malaria journal* 11.1 (2012): 1–15. doi: 10.1186/1475-2875-11-328 22985344 PMC3489857

[3] Pavli Androula, and Maltezou Helena C.. "Malaria and travellers visiting friends and relatives." *Travel medicine and infectious disease* 8.3 (2010): 161–168. doi: 10.1016/j.tmaid.2010.01.003 20541136

[4] Xie Yiting, et al. "Molecular epidemiological surveillance of Africa and Asia imported malaria in Wuhan, Central China: comparison of diagnostic tools during 2011–2018." *Malaria Journal* 19.1 (2020): 1–14. 32883296 10.1186/s12936-020-03387-2 PMC7470674

[5] Lai S., Wardrop N. A., Huang Z., Bosco C., Sun J., Bird T., et al. (2016). *Plasmodium falciparum* malaria importation from Africa to China and its mortality: an analysis of driving factors. *Scientific reports*, 6(1), 1–9 28000753 10.1038/srep39524 PMC5175130

[6] Zhang Qian, et al. "The epidemiology of Plasmodium vivax and Plasmodium falciparum malaria in China, 2004–2012: from intensified control to elimination." *Malaria Journal* 13.1 (2014): 1–9. 25363492 10.1186/1475-2875-13-419 PMC4232696

[7] Zhou Sheng, et al. "Trends of imported malaria in China 2010–2014: analysis of surveillance data." *Malaria Journal* 15.1 (2016): 1–8. 10.1186/s12936-016-1093-0 PMC4727325 26809828

[8] Li Zhongjie, et al. "Epidemiologic features of overseas imported malaria in the People’s Republic of China." *Malaria Journal* 15.1 (2016): 1–9. 26946150 10.1186/s12936-016-1188-7 PMC4779568

[9] Sun Q., Yang Y., Xiao N., Zhou S., Lin K., Wang D., et al. Malaria Imported from Ghana by Returning Gold Miners, China, 2013." *Emerging infectious diseases* 21.5 (2015): 864. doi: 10.3201/2105.141712 25897805 PMC4412230

[10] Liu Yaobao, et al. "Malaria in overseas labourers returning to China: an analysis of imported malaria in Jiangsu Province, 2001–2011." *Malaria Journal* 13.1 (2014): 1–9. doi: 10.1186/1475-2875-13-29 24460982 PMC3922785

[11] De Wu Z. D., Lin R., Mao Q., Lu W., Ruan C., Cen Y., et al. (2021). Malaria Surveillance of Entry People During the COVID-19 Epidemic—Guangdong Province, China, October 2020–May 2021. *China CDC Weekly*, Sep 17; 3(38), 799–802. doi: 10.46234/ccdcw2021.180 . 34594993 PMC8477056

[12] Wang X., He Z., Wu W., Liang Z., Li X., Ren H., et al. (2021). Screening and diagnosis of two imported malaria cases under the situation of epidemic prevention and control of COVID-19. *Acta Parasitologica et Medica Entomologica Sinica*, 71–75.

[13] Jian-hai Y. I. N., Li Z. H. A. N. G., Hong T. U., He-jun Z. H. O. U., & Zhi-gui X. I. A. (2021). Analysis of case-based malaria surveillance and response during the period of COVID-19 outbreak in China. *Chinese Journal of Parasitology and Parasitic Diseases*, 39(4), 461.

[14] Rahmadani F., & Lee H. (2020). Hybrid deep learning-based epidemic prediction framework of COVID-19: South Korea case. *Applied Sciences*, 10(23), 8539.

[15] Zhang G., & Liu X. (2021). Prediction and control of COVID-19 spreading based on a hybrid intelligent model. *Plos one*, 16(2), e0246360. doi: 10.1371/journal.pone.0246360 33571234 PMC7877772

[16] Verma H., Mandal S., & Gupta A. (2022). Temporal deep learning architecture for prediction of COVID-19 cases in India. *Expert Systems with Applications*, 116611. doi: 10.1016/j.eswa.2022.116611 35153389 PMC8817764

[17] Absar N., Uddin N., Khandaker M. U., & Ullah H. (2022). The efficacy of deep learning based LSTM model in forecasting the outbreak of contagious diseases. Infectious Disease Modelling, 7(1), 170–183. doi: 10.1016/j.idm.2021.12.005 34977438 PMC8712463

[18] CDC Digital Repository. China disease prevention and control center for infectious disease prevention and control. data from: Chinese center for disease control and prevention, 2019. https://www.phsciencedata.cn/

[19] Feng J., Tu H., Zhang L., Xia Z., & Zhou S. (2020). Imported Malaria Cases—China, 2012–2018. *China CDC Weekly*, 2(17), 277. doi: 10.4269/ajtmh.14-0733 34594639 PMC8422169

[20] Zhang L., Tu H., Zhou S., Xia Z., & Feng J. (2021). Malaria Deaths—China, 2011–2020. *China CDC Weekly*, 3(17), 360. doi: 10.46234/ccdcw2021.098 34594884 PMC8392890

[21] Khandelwal I., Adhikari R., and Verma G. (2015). Time series forecasting using hybrid ARIMA and ANN models based on DWT decomposition. Procedia Computer Science, 48(1):173–179.

[22] Zhang R., Song H., Chen Q., Wang Y., Wang S., & Li Y. (2022). Comparison of ARIMA and LSTM for prediction of hemorrhagic fever at different time scales in China. Plos one, 17(1), e0262009. doi: 10.1371/journal.pone.0262009 35030203 PMC8759700

[23] Panigrahi S. and Behera H. (2017). A hybrid ETS-ANN model for time series forecasting. Engineering Applications of Artificial Intelligence, 66:49–59.

[24] Jing-diao C. H. E. N., Rong-xing L. I. N., Zhuo-hui D. E. N. G., Bo P. A. N., Fu-quan P. E. I., Wen-cheng L. U., et al. (2020). Analysis on the epidemic situation of imported malaria in Guangdong Province from 2011 to 2019. *Journal of Tropical Diseases and Parasitology*, 18(4), 197.

[25] Yang H. L., Baloch Z., Xu J. W., Sun X. D., Lin Z. R., Zhou Y. W., et al. (2021). Malaria: elimination tale from Yunnan Province of China and new challenges for reintroduction. *Infectious Diseases of Poverty*, 10(04), 86–89. doi: 10.1186/s40249-021-00866-9 34289905 PMC8293506

[26] Ding C., Huang C., Zhou Y., Fu X., Liu X., Wu J., et al. (2020). Malaria in China: a longitudinal population-based surveillance study. *Epidemiology & Infection*, 148. 10.1017/S0950268820000333 PMC7058654 32089144

[27] Lin H., Lu L., Tian L., Zhou S., Wu H., Bi Y., et al. (2009). Spatial and temporal distribution of falciparum malaria in China. *Malaria Journal*, 8(1), 1–9. doi: 10.1186/1475-2875-8-130 19523209 PMC2700130

[28] Noda H. (2022). A Model to Estimate the Effect of International Traffic on Malaria Cases: The Case of Japan from 1999 to 2021. *International Journal of Environmental Research and Public Health*, 19(2), 880. doi: 10.3390/ijerph19020880 35055702 PMC8775555

